# Expression profile of messenger and micro RNAs related to the histaminergic system in patients with five subtypes of breast cancer

**DOI:** 10.3389/fonc.2024.1407538

**Published:** 2024-08-29

**Authors:** Tomasz Sirek, Agata Sirek, Marcin Opławski, Dariusz Boroń, Michał Chalcarz, Piotr Ossowski, Konrad Dziobek, Nikola Zmarzły, Damian Strojny, Beniamin Oskar Grabarek

**Affiliations:** ^1^ Department of Plastic Surgery, Faculty of Medicine, Academia of Silesia, Katowice, Poland; ^2^ Department of Plastic and Reconstructive Surgery, Hospital for Minimally Invasive and Reconstructive Surgery in Bielsko-Biała, Bielsko-Biala, Poland; ^3^ Department of Medical and Health Sciences, Collegium Medicum, WSB University, Dabrowa Górnicza, Poland; ^4^ Department of Gynecology and Obstetrics with Gynecologic Oncology, Ludwik Rydygier Memorial Specialized Hospital, Kraków, Poland; ^5^ Department of Gynecology and Obstetrics, Faculty of Medicine and Health Sciences, Andrzej Frycz Modrzewski University in Kraków, Kraków, Poland; ^6^ Uczelnia Medyczna im, Marii Skłodowskiej-Curie, Warszawa, Poland; ^7^ Chalcarz Clinic-Aesthetic Surgery, Aesthetic Medicine, Poznan, Poland; ^8^ Bieńkowski Medical Center-Plastic Surgery, Bydgoszcz, Poland; ^9^ Institute of Health Care, National Academy of Applied Sciences in Przemyśl, Przemyśl, Poland; ^10^ Department of Medical Science, New Medical Techniques Specjalist Hospital of St. Family in Rudna Mała, Rzeszów, Poland; ^11^ Department of Molecular, Biology Gyncentrum Fertility Clinic, Katowice, Poland

**Keywords:** breast cancer, histaminergic system, therapeutic targets, mRNA, miRNA, microarray, ELISA

## Abstract

Disparities in estrogen receptor (ER), progesterone receptor, human epidermal growth factor receptor 2 (HER2), and Ki67 proliferation indices facilitate the categorization of breast cancer into four principal subtypes: luminal A, luminal B, HER2-positive, and triple-negative breast cancer (TNBC). Preclinical studies investigating the therapeutic potential of histaminergic system targeting in breast cancer have shown promising results. This study aimed to assess the expression profiles of messenger ribonucleic acid (mRNA) and micro RNA (miRNA) related to the histaminergic system in five subtypes of breast cancer among Polish women. Patients with five breast cancer subtypes were included in the study: luminal A (n = 130), luminal B (n = 196, including HER2-, n =100; HER2+, n= 96), HER2+ (n = 36), and TNBC (n = 43). They underwent surgery during which the tumor tissue was removed along with a margin of healthy tissue (control material). Molecular analysis included the determination of a microarray profile of mRNAs and miRNAs associated with the histaminergic system, real-time polymerase chain reaction preceded by reverse transcription of selected genes, and determination of histamine receptors (human histamine H1 receptor [HRH1], human histamine H2 receptor [HRH2], and human histamine H4 receptor [HRH4]) using an enzyme-linked immunosorbent assay. Statistical analysis was performed with statistical significance at p < 0.05. Nine mRNAs were significantly differentiated in breast cancer sections, regardless of subtype, compared to control samples: *HRH1, HRH2*, *HRH4*, histamine N-methyltransferase *(HNMT)*, 5-hydroxytryptamine receptor 6 (*HTR6)*, endothelin 1 (*EDN1)*, endothelin receptor type A *(EDNRA)*, adenosine deaminase *(ADA)*, solute carrier family 22 member 3 (*SLC3A2).* Predictive analysis showed that hsa-miR-34a potentially regulates *HRH1* expression, whereas hsa-miR-3140-5p and hsa-miR-4251 potentially affect *HRH2* expression. In contrast, *HRH4* and *EDN1* expression were regulated by hsa-miR-1-3p. The expression of *HNMT* is potentially regulated by one miRNA, hsa-miR-382, whereas *EDNRA* expression is regulated by two miRNA molecules: hsa-miR-34a and hsa-miR-16. In contrast, hsa-miR-650 is involved in the regulation of *HTR6* expression, whereas hsa-miR-1275 potentially interacts with three mRNAs: *ADA*, *SLC23A2*, and *HRH1*. Molecular analysis confirmed that the selected mRNA and miRNA transcripts could be promising molecular markers and therapeutic targets.

## 1 Introduction

Breast cancer is the most frequently diagnosed malignancy among women, as indicated by the World Health Organization (WHO) in 2020 (1). In Poland, cancer is the second leading cause of death, with malignant tumors being the primary cause of mortality among women aged <65 years (2).

Breast cancer is a heterogeneous disease with diverse molecular and clinicopathological characteristics. Categorization into four principal subtypes (luminal A, luminal B, human epidermal growth factor receptor 2-positive [HER2+], and triple-negative breast cancer [TNBC]) is facilitated by disparities in estrogen receptor (ER), progesterone receptor (PgR), HER2, and the Ki67 proliferation index. Each subtype exhibits distinct prognostic implications, necessitating tailored therapeutic approaches (3–5).

The luminal A subtype, characterized by ER and PgR positivity and a low Ki67 index, typically shows a favorable prognosis and responds well to hormone therapy. The luminal B subtype is further divided into HER2-negative (HER2**−)** and HER2+ cohorts, both expressing ER, but differing in HER2 expression and proliferation rates. Human epidermal growth factor receptor 2-positive breast cancer is characterized by the overexpression of HER2 protein, leading to aggressive tumor growth and necessitating anti-HER2 therapy. Triple-negative breast cancer, which lacks ER, PgR, and HER2 expression, is known for its aggressive nature and poor prognosis, and requires chemotherapy and targeted treatment (6–10).

Despite advancements in breast cancer treatments, several limitations persist. Hormone therapies that are effective against luminal subtypes may lead to resistance over time. Anti-HER2 therapies, although improving outcomes in HER2+ patients, they can cause significant side effects and may not be effective in all patients (11–14). Additionally, TNBC lacks targeted therapies, often resulting in poor outcomes owing to the reliance on chemotherapy alone (15, 16). These challenges highlight the need for novel therapeutic strategies to improve efficacy and reduce adverse effects.

Histaminergic systems, traditionally associated with allergic responses and neurotransmission, have recently gained attention for their potential roles in breast cancer. Histamine, a biogenic amine, interacts with four G-protein-coupled receptors (human histamine H1 receptor [HRH1], human histamine H2 receptor [HRH2], human histamine H3 receptor [HRH3], and human histamine H4 receptor [HRH4]) and influences various physiological processes. Emerging evidence suggests a complex interplay between histamine levels and breast cancer progression. Histamine receptors, particularly HRH1 and HRH2, have been implicated in tumor growth, angiogenesis, invasion, and metastasis. Changes in histamine levels and receptor expression in breast cancer tissues compared with normal tissues suggest a role for the histaminergic system in cancer development (17–24).

The histaminergic system, initially recognized for its involvement in allergic responses and neurotransmission, has recently attracted attention for its potential role in breast cancer (25–29). Pharmacological agents targeting histamine receptors or synthesis have shown anti-tumor effects, underscoring the therapeutic significance of this pathway​. Despite promising outcomes, the precise mechanisms underlying the involvement of the histaminergic system in breast cancer remain to be fully understood (26, 27, 30–33).

The novelty of this study lies in the comprehensive molecular analysis of the histaminergic system in various breast cancer subtypes, an area that has not yet been extensively explored. By using advanced techniques, such as microarray profiling, quantitative reverse transcription polymerase chain reaction (RT-qPCR), and enzyme-linked immunosorbent assay (ELISA), this study provides a detailed expression profile of histamine-related mRNAs and miRNAs. These findings could pave the way for new therapeutic strategies targeting the histaminergic system, thereby offering potential improvements in the treatment and management of breast cancer. Additionally, the focus of this study on a Polish cohort adds valuable regional data to our global understanding of breast cancer biology.

This study aimed to assess the expression profiles of messenger mRNAs and microRNAs (miRNAs) related to the histaminergic system in five breast cancer subtypes in Polish women. By identifying the differentially expressed genes and miRNAs, this study aimed to identify potential molecular markers and therapeutic targets within the histaminergic pathway.

## 2 Materials and methods

### 2.1 Ethics

This study adhered to the 2013 Declaration of Helsinki guidelines for human experimentation. This study was approved by the Bioethical Committee of the Regional Medical Chamber in Krakow (No. 81/KBL/OIL/2023, dated 10 March 2023). Data confidentiality and anonymity were maintained at all times. Patient identification information was deleted before analyzing the database. Identifying patients individually was impossible, either from this study or from the database.

### 2.2 Participants

Patients with five breast cancer subtypes were included in the study: luminal A (n = 130), luminal B (n = 196, including HER2**−** n =100; HER2+ n= 96), HER2+ (n = 36), and TNBC (n = 43). They underwent surgery, during which the tumor tissue was removed along with a margin of healthy tissue (control material). Characteristics of the patients with each of the five subtypes are shown in **Table 1**. According to the tumor, nodule, and metastasis (TNM) classification, all the patients were classified as T1N0M0 (34). Inclusion and exclusion criteria are shown in **Table 2**.

**Table 1:** Characteristics of patients.

Molecular type	Degree of histological malignancy			Age	
	G1	G2	G3	<50 years	>50 years
Luminal A	23 (18%)	48 (37%)	59 (45%)	43 (33%)	87 (67%)
Luminal B HER2-	31 (31%)	57 (57%)	12 (12%)	32 (32%)	68 (68%)
Luminal B HER+	23 (24%)	57 (59%)	16 (17%)	19 (20%)	77 (80%)
Non-luminal HER2+	9 (25%)	12 (33%)	15 (42%)	9 (25%)	27 (75%)
TNBC	14 (32%)	21 (49%)	8 (19%)	10 (23%)	33 (77%)

Data are presented as number of cases and (percentage); human epidermal growth factor receptor 2; TNBC, triple-negative breast cancer.

**Table 2:** Inclusion and exclusion criteria.

Inclusion criteria	Exclusion criteria
Expressing informed, voluntary consent to participate in the study	Failure to express informed, voluntary consent to participate in the study
Patients diagnosed with one of the five breast cancer subtypes (luminal A, luminal B HER2-, luminal B HER2+, non-luminal HER2+, and TNBC)	Patients with a history of other malignancies
Patients who underwent surgical removal of the tumor along with a margin of healthy tissue	Patients who received neoadjuvant chemotherapy or radiotherapy prior to surgery.
Patients aged between 18 and 75 years	Patients aged below 18 and over 75 years
Patients classified as T1N0M0 according to TNM classification	Patients with metastatic disease (stages II-IV)

### 2.3 Total RNA extraction

Ribonucleic acid was extracted from tissues using the TRIzol reagent (Invitrogen Life Technologies, Carlsbad, CA, USA; catalog number: 15596026), according to the manufacturer’s protocol. Subsequently, the isolated RNA was purified using the RNeasy Mini Kit (QIAGEN, Hilden, Germany; catalog number: 74104) and treated with DNase I (Fermentas International Inc., Burlington, ON, Canada; catalog number: 18047019). Qualitative evaluation of RNA was performed using 1% agarose gel electrophoresis with 0.5 mg/mL ethidium bromide, while quantitative assessment was conducted by measuring absorbance at 260 nm.

### 2.4 Microarray profiling of histaminergic system-related genes

The differential expression of histaminergic system-related genes in tumor tissues compared to control tissues was analyzed using the HG-U 133_A2 microarray (Affymetrix, Santa Clara, CA, USA) and GeneChip™ 3′ IVT PLUS reagent kit (Affymetrix, Santa Clara, CA, USA; catalog Number 902416), according to the manufacturer’s instructions and methods outlined in previous studies. Of 22,277 mRNA probes identified on the microarray plate, 65 were associated with the histaminergic system. These 65 mRNAs were selected by entering the term “histaminergic system” into the Affymetrix NetAffx Analysis Center database, which provided a list of mRNAs known to be related to the histaminergic system.

Microarray analysis involved the synthesis of double-stranded complementary DNA (cDNA) using a GeneChip 30IVT Express kit, followed by RNA amplification and fragmentation. Subsequently, the artificial RNAs were hybridized, and fluorescence intensity was measured using an Affymetrix Gene Array Scanner 3000 7G and Gene Chip^®^ Command Console^®^ Software (Affymetrix, Santa Clara, CA, USA).

### 2.5 Microarray profiling of histaminergic system-related miRNAs and its potential influence on the expression of analyzed genes

Microarray profiles of miRNAs were analyzed using the commercially available GeneChip miRNA 2.0 Array (Affymetrix), following the manufacturer’s instructions.

### 2.6 Analysis of the expression profile of selected genes via RTq-PCR

Validation of the microarray data was performed using RT-qPCR with the SensiFast SYBR No-ROX One-Step kit (Bioline, London, UK), according to the manufacturer’s instructions. Expression patterns of selected genes were presented using the 2^−ΔΔCt^ method. Beta actin (*ACTB*) served as an endogenous control. The thermal cycling conditions were as follows: reverse transcription at 45°C for 10 min, polymerase activation at 95°C for 2 min, followed by 40 cycles of denaturation at 95°C for 5 s, annealing at 60°C for 10 s, and extension at 72°C for 5 s. The primer sequences are presented in **Table 1** in the **Supplementary Material**. Each reaction was performed in triplicate.

### 2.7 Evaluation of the expression profile of miRNAs via RTq-PCR

In this step, we validated the expression patterns of 17 differentially expressed miRNAs using RT-qPCR. Reverse transcription was performed using 10 ng of total RNA in a 15-μL reaction volume with the TaqMan MicroRNA Reverse Transcription Kit (Thermo Fisher Scientific, Waltham, MA, USA), according to the manufacturer’s instructions. Next, miRNA quantification was performed using TaqMan MicroRNA Assays (Thermo Fisher Scientific, Waltham, MA, USA) for hsa-miR-34a (Assay ID 000426), hsa-miR-3140-5p (Assay ID 462283_mat), hsa-miR-4251 (Assay ID 243741_mat), hsa-miR-1-3p (Assay ID 002222), hsa-miR-382 (Assay ID 000572), hsa-miR-16 (Assay ID 000391), hsa-miR-650 (Assay ID 001603), and hsa-miR-1275 (Assay ID hsa-miR-1275). The thermal profile of the reaction was as follow: initial denaturation at 95°C for 10 min, followed by 40 cycles at 95°C for 15 s and 60°C for 1 min. Each reaction was performed in triplicate, and RNU48 served as an endogenous control.

### 2.8 ELISA analysis

The final part of this study involved assessing alterations in HRH1-4 expression in cancer and control samples using ELISA. To determine the protein concentrations, we used the following ELISA kits, according to the manufacturer’s instructions: HRH1 ELISA Kit (MyBioSource, Inc., San Diego, CA 92195-3308, USA; catalog number MBS454215), HRH2 ELISA Kit (MyBioSource, Inc., San Diego, CA 92195-3308, USA; catalog number MBS9355797), and HRH4 ELISA Kit (MyBioSource, Inc., San Diego, CA 92195-3308, USA; catalog number MBS2023167).

### 2.9 Statistical analysis

Statistical analysis of the microarray results was conducted using Transcriptome Analysis Console programs (Affymetrix, Santa Clara, CA, USA). Statistical analyses of the RT-qPCR and ELISA results were performed using Statistica 13.0 PL (StatSoft, Cracow, Poland).

#### 2.9.1 Statistical analysis of mRNA microarray results

Statistical analysis of the oligonucleotide microarray results was performed using MicroArray Suite 5.0 (Affymetrix, Santa Clara, CA, USA) and a data mining tool (Affymetrix, Santa Clara, CA, USA). Normalization of the results using the robust multi-array average (RMA) method, which involves logarithmic transformation of the fluorescence signal values for each transcript (log_2_), was performed using RMA Express (Affymetrix, Santa Clara, CA, USA). Statistical analyses were performed using the Transcriptome Analysis Console program (Affymetrix, Santa Clara, CA, USA). Samples were clustered using hierarchical clustering with Euclidean distance measurements. Based on the data in the Affymetrix NetAffxTM database, 65 mRNA IDs were selected using the query “histaminergic system” (accessed February 19, 2024), which can be analyzed using oligonucleotide microarrays with HGU133A_2.0 plates. The analysis included fluorescence values for all types of probes, such as “_at” (complementary to the sequence of a specific transcript), “_s_at” (matching different transcripts, various polyadenylation products, or alternative splicing forms), and “_x_at” (complementary to similar but not identical sequences, hybridizing with sequences similar to the target). The fold-change (FC) parameter was used to assess the magnitude of the difference in mRNA expression levels between compared transcriptome groups, and the p value assessed the significance of the observed difference. A gene was considered differentially expressed if the absolute fold change in the fluorescence signal between the compared samples was >1.1 (at least a 1.1-fold increase or decrease in signal intensity) and the p value <0.05. Gene differentiation between the studied transcriptome groups was determined using one-way analysis of variance (ANOVA) with Benjamini–Hochberg correction and Tukey’s *post-hoc* test.

#### 2.9.2 Statistical analysis of miRNA microarray results

The miRNA microarray results were statistically analyzed using miRNAQC Tool version 1.1.10 (Affymetrix, Santa Clara, CA, USA) and Transcriptome Analysis Console 2.0 (Affymetrix, Santa Clara, CA, USA; February 19, 2024). Initial raw data from the GeneChip miRNA 2.0 Array were background-corrected using the miRNAQC Tool to remove noise and enhance signal accuracy, ensuring that the measured intensities reflected true biological variation rather than technical artifacts. Corrected data were normalized using the RMA method implemented in the miRNA QC tool. This process included quantile normalization to ensure that the distributions of probe intensities were identical across all arrays, thereby minimizing inter-array variability. After normalization, the fluorescence signal intensity of each miRNA was logarithmically transformed (log_2_). The FC parameter was used to assess the magnitude of the difference in miRNA expression levels between the compared transcriptome groups, while the p-value was used to assess the significance of the observed difference. An miRNA was considered differentiated if the absolute fold change in the fluorescence signal between the compared samples was >1.1 (at least a 1.1-fold increase or decrease in signal intensity) and the p value was <0.05. Micro RNA differentiation between the studied transcriptome groups was determined using one-way ANOVA with Benjamini–Hochberg correction and Tukey’s *post-hoc* test. Differentially expressed miRNAs between tumor and control tissues involved in regulating differentially expressed mRNAs were determined using the TargetScan database (http://www.targetscan.org/) (35) and miRanda (http://mirdb.org) (36). A predicted target with a score exceeding 80 was deemed highly likely to be authentic. However, caution was advised if the score falls below 60, and additional supporting evidence was recommended (36, 37).

#### 2.9.3 Statistical analysis of RTqPCR and ELISA results

The results were entered into a Microsoft Excel spreadsheet to create a database, which was then implemented using the licensed version of Statistica 13.0 PL (StatSoft, Cracow, Poland) for statistical calculations. Commonly accepted significance level in medical research (p < 0.05) was used for the statistical analysis. The hypothesis regarding the normal distribution of a parameter was verified using the Shapiro–Wilk test. The hypothesis regarding The homogeneity of variance was verified using the Levene’s test. Given that the assumption of normality was satisfied, further analyses were conducted using parametric methods. Differences between groups concerning individual variables were initially verified using one-way ANOVA, and the nature of specific relationships was determined using the Tukey’s *post-hoc* test.

#### 2.9.4 Statistical analysis of search tools for the retrieval of interacting genes/proteins results

Relationships between genes were thoroughly examined using the STRING Database 11.0 (accessed July 3, 2024). Within the STRING database, the parameter “strength” (Log10 [observed/expected]) quantifies the extent of the enrichment effect. This parameter reflects the ratio between the following: (1) the number of proteins annotated with a specific term within the network; and (2) the expected number of proteins annotated with that term in a randomly generated network of equivalent size. Conversely, the false discovery rate parameter assesses the significance of the enrichment, with p values adjusted for multiple testing within each category using the Benjamini–Hochberg procedure (38).

#### 2.9.5 Overall survival analysis

The Kaplan–Meier plotter (http://kmplot.com/; accessed July 3, 2024) was used to plot the overall survival status in every group for the nine mRNAs that significantly differentiated breast cancer, regardless of subtype, compared to the control. The follow-up threshold was 60 months (39, 40).

## 3 Results

### 3.1 Microarray profile of histaminergic system-related gene breast cancer samples compared with control tissue

A one-way ANOVA was conducted in the first step to determine the differential expression of mRNAs associated with the histaminergic system. We used the Affymetrix NetAffx Analysis Center database to identify mRNAs specific to the histaminergic system. This database allowed the filtering and selection of 65 mRNAs associated with the histaminergic system. Our analysis indicated that 17 of these mRNAs significantly differentiated cancer samples from the controls (p < 0.05).

Next, we applied the Tukey’s *post-hoc* test to further analyze these 17 mRNAs, identifying which mRNAs specifically differentiated between breast cancer subtypes and control samples. This test also highlighted the mRNAs common to more than one breast cancer subtype.

Nine mRNAs were significantly differentiated in breast cancer sections, regardless of subtype, compared to the control samples: *HRH1, HRH2*, *HRH4*, histamine N-methyltransferase (*HNMT*), 5-hydroxytryptamine receptor 6 (*HTR6*), endothelin 1 (*EDN1*), endothelin receptor type A (*EDNRA*), adenosine deaminase (*ADA*), solute carrier family 22 member 3 (*SLC3A2*).

**Figure 1:**
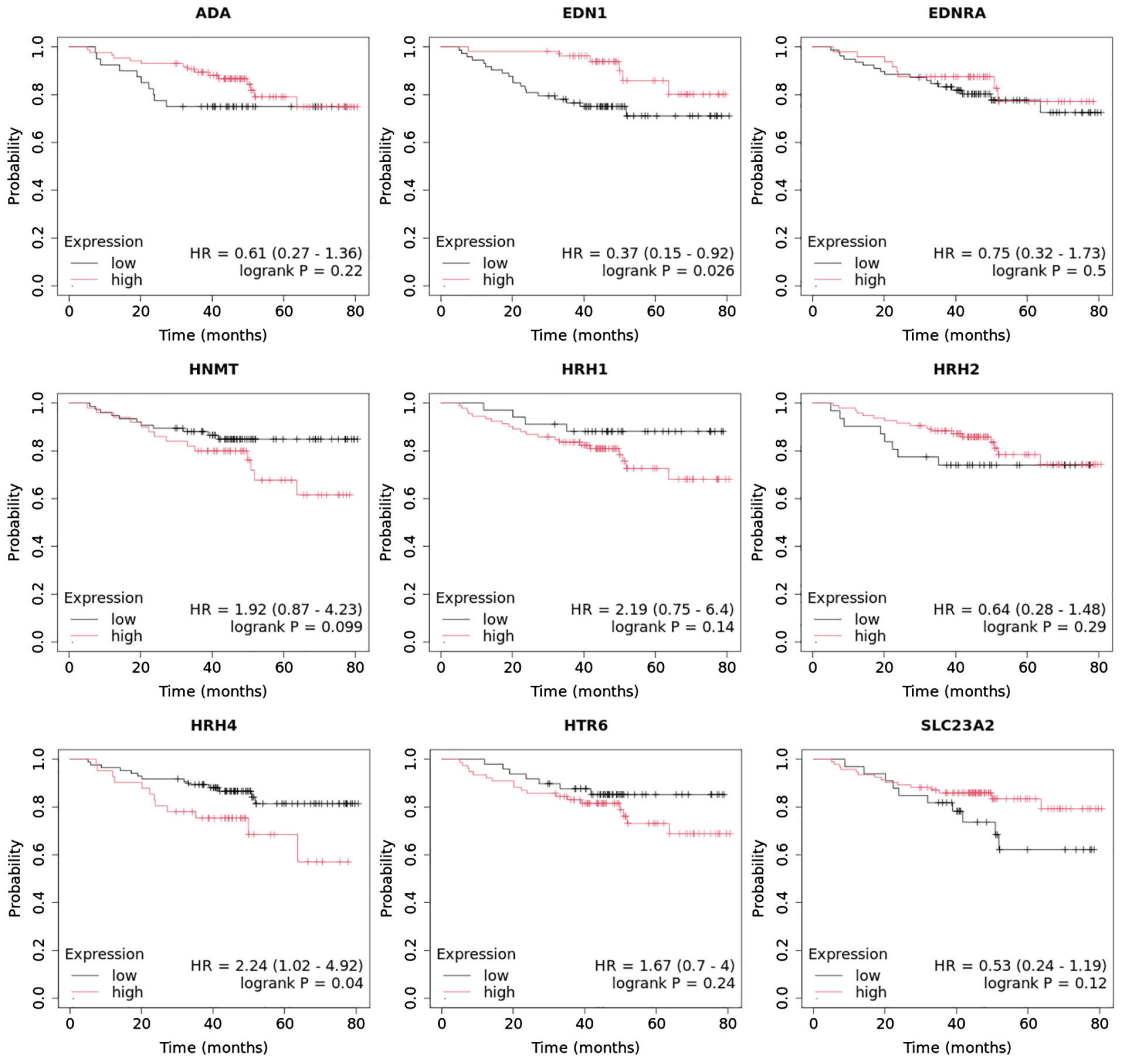
Venn diagram of microarray results (p<0.05).

**Figure 2:**
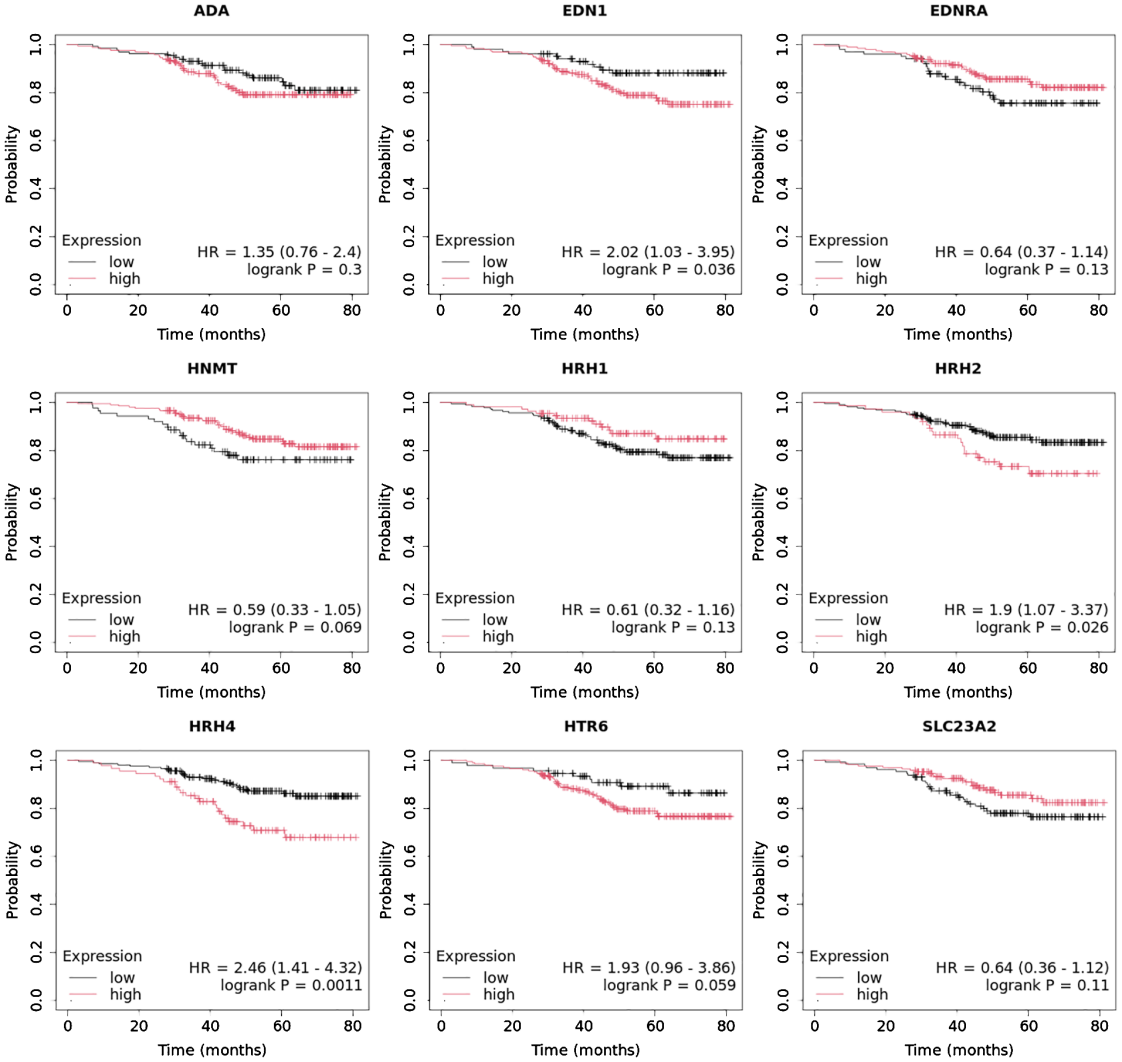
Changes in the expression profile of genes differentiating tumor samples compared to control tissues obtained by microarray analysis.

In contrast, the three mRNAs that showed statistically significant changes in expression were specific for luminal subtype A and corresponded to gamma-aminobutyric acid type A receptor subunit beta 1 (*GABRB1*), adenylate cyclase-activating polypeptide 1 (*ADCYAP1*) and Sorting Nexin 1 (*SNX*). For the luminal B HER2**−,** HER2+, and non-luminal HER2+ subtypes, no mRNA was shown to be specific. Thus, gonadotropin-releasing hormone 2 (*GNRH2*) and 5-hydroxytryptamine receptor 2 B (*HTR2B*) were common subtype B tumors, depending on the presence of HER2 on the cell surface. In contrast, the regulators of G-protein signaling (*RGS4*), period circadian clock 2 (*PER2*), LYN proto-oncogene, and Src family tyrosine kinase (*LYN*) genes were characteristic of the TNBC subtype. A Venn diagram was created to identify the genes shared among all the transcriptome groups and those exclusive to specific groups (**Figure 1**). The expression profile of the selected 17 genes in different subtypes of breast cancer compared to the control samples are presented in **Figure 2**.

### 3.2 Results of selected expression of mRNAs via RTqPCR

**Figure 3:**
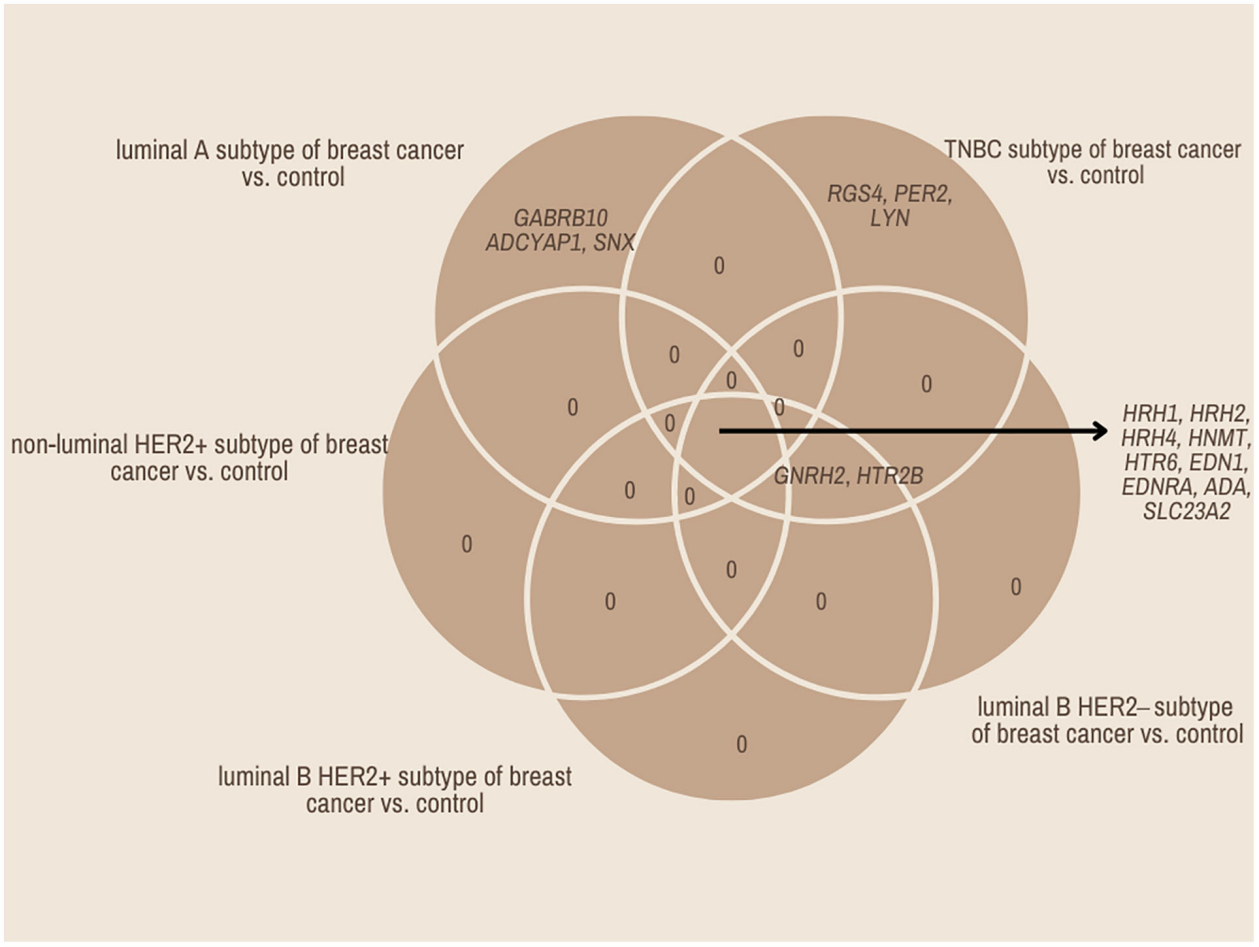
Changes in expression profile of mRNAs in different subtypes of breast cancer samples compared to control samples obtained via RTqPCR.

Next, we evaluated the changes in the expression patterns of the 17 genes selected in the microarray experiment that differentiated individual breast cancer subtypes from controls by performing RT-qPCR (validation). Quantitative RT-qPCR analysis confirmed the expression changes indicated by the semi-quantitative microarray analysis for all mRNAs. Regardless of breast cancer subtype, overexpression of the following mRNAs was observed: *HRH1*, *HRH2*, *HRH4*, *HNMT*, *EDN1*, *EDNRA*, *HTR6*, *GABRB1*, *ADCYAP1*, *ADA*, and *SLC23A2*. In turn, the expression profiles of mRNA, *GNRH2*, and *PER2* were reduced in the tumor tissues compared to the controls. In contrast, the expression patterns of *SNX, HTR2B, RGS4*, and *LYN* were not homogeneous and differed among the breast cancer subtypes (**Figure 3**).

### 3.3 Expression patterns of miRNAs obtained via the microarray analysis

**Figure 4:**
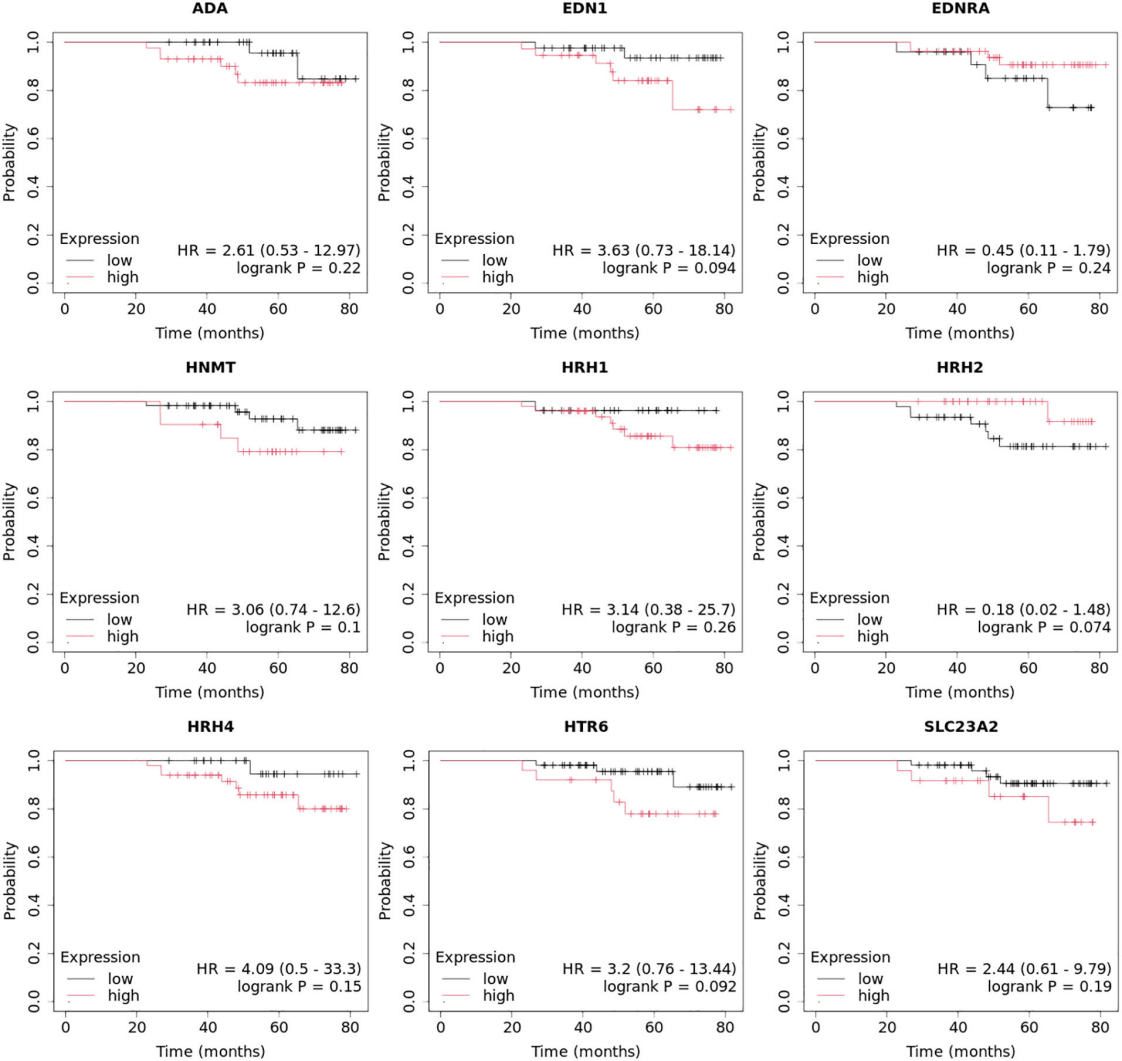
All miRNAs affecting the transcriptional activity of genes in all different subtypes of breast cancer compared to control samples.

**Figure 5:**
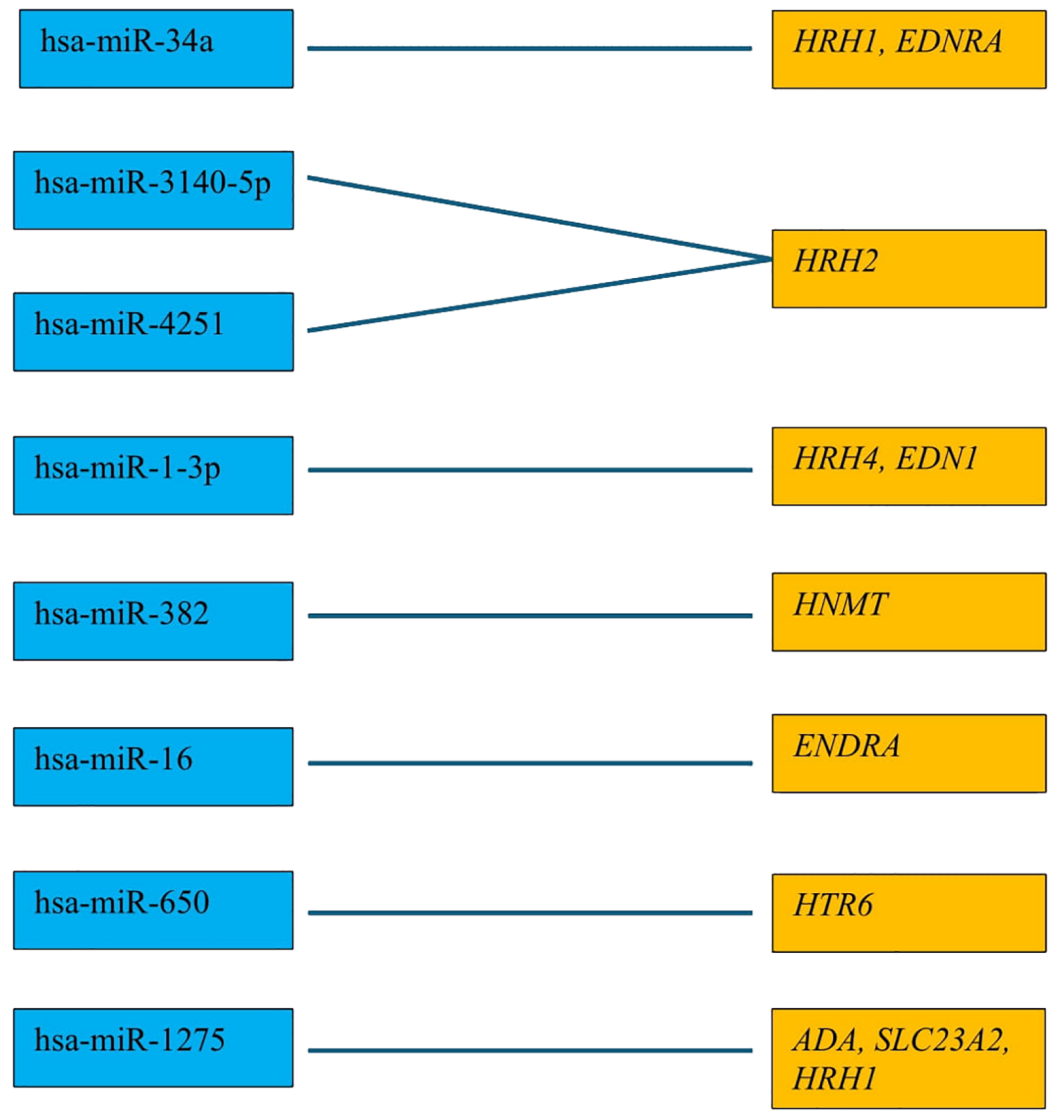
Expression profile of miRNAs potentially regulated selected mRNA in different subtypes of breast cancer tissues in comparison to control tissues obtained by microarray analysis.

Next, we evaluated which of the miRNAs whose expression was significantly different in each breast cancer subtype compared to the control (p < 0.05) was involved in the post-transcriptional regulation of the previously selected differentiating mRNAs in the microarray experiment. For mRNAs that differentiated tumor tissues independent of breast cancer subtype, we looked for miRNAs whose expression occurred in all subtypes, and the condition that the predicted target with a prediction score exceeding 80 was met. For mRNAs that specifically differentiated between two or only one breast cancer subtype, miRNA expression was reported only in breast cancer subtype A, where predictive analysis showed that hsa-miR-34a potentially regulated *HRH1* expression, and hsa-miR-3140-5p and hsa-miR-4251 potentially affected *HRH2* expression. In contrast, *HRH4* and *EDN1* expression were regulated by hsa-miR-1-3p. The expression of *HNMT* is potentially regulated by hsa-miR-382, whereas *EDNRA* expression is regulated by two miRNAs: hsa-miR-34a and hsa-miR-16. In contrast, hsa-miR-650 is involved in the regulation of *HTR6* expression, whereas hsa-miR-1275 potentially interacts with three mRNAs: *ADA*, *SLC23A2*, and *HRH1*. Additionally, for the *GABRB1*, *HTR2B*, *RGS4*, and *PER* genes, the regulatory effect of miRNAs was demonstrated. In contrast, predictive analysis did not confirm whether *ADYAP1*, *SNX*, or *GNRH2* expression was regulated by the selected differentially expressed miRNAs (**Figure 4**). The expression profiles of miRNAs in all subtypes of breast cancer compared to control samples are presented in **Figure 5**.

#### 3.3.1 Expression patterns of miRNA expression obtained via RTqPCR

**Figure 6:**
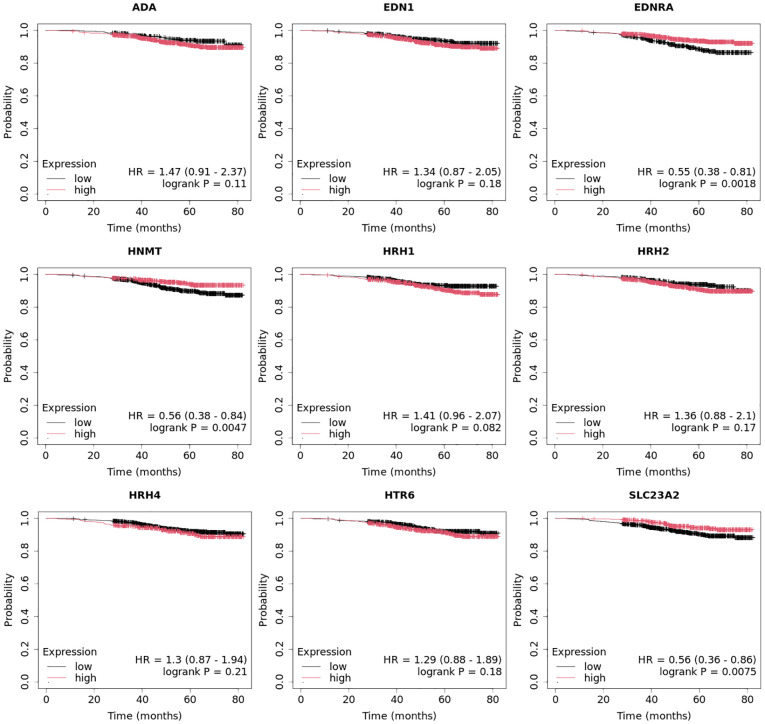
Expression profile of miRNAs potentially regulated selected mRNA in different subtypes of breast cancer tissues in comparison to control tissues obtained by RTqPCR analysis.

We validated the results obtained using qRT-PCR for the miRNAs selected in the microarray experiments. For all the miRNAs analyzed by qRT-PCR, the same direction of expression changes, as indicated by semi-quantitative microarray analysis, was obtained (**Figure 6**).

### 3.4 Expression profile of *HRH1*, *HRH2*, and *HRH4* in breast cancer tissues and controls at the protein level

**Figure 7:**
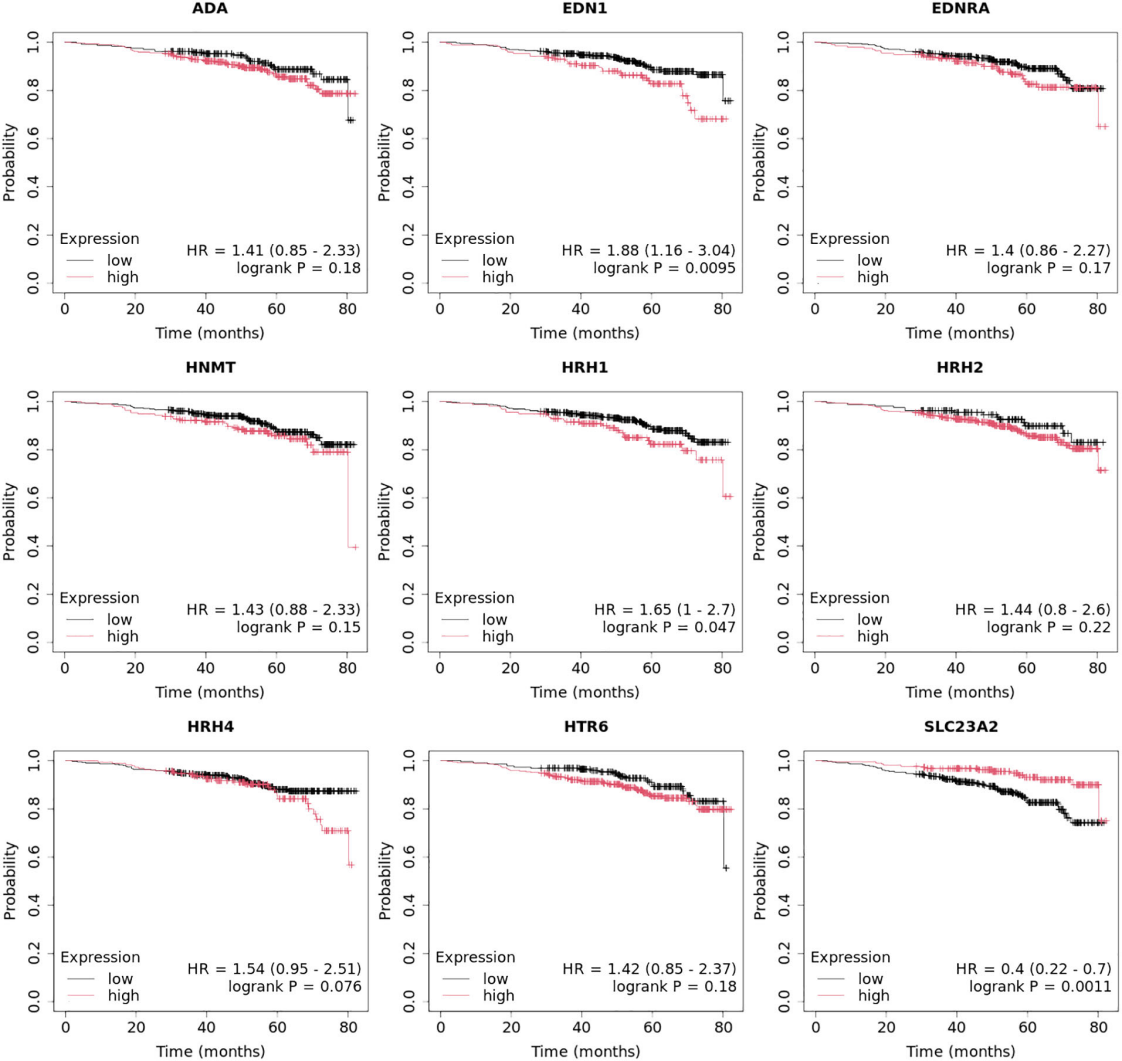
Concentration of HRH1, HRH2, and HRH4 in different subtypes of breast cancer and control tissue.

At the protein level, we observed significantly higher concentrations of HRH1, HRH2, and HRH4 in tumor tissues than those in control tissues (**Figure 7**; p < 0.05). The highest concentrations of HRH1 and HRH2 receptors at the protein level were found in the TNBC samples. In contrast, the luminal B HER2**−** subtype showed the highest HRH4 levels (**Figure 7**).

### 3.5 Relationship network for the selected histaminergic system differentiation genes

**Figure 8:**
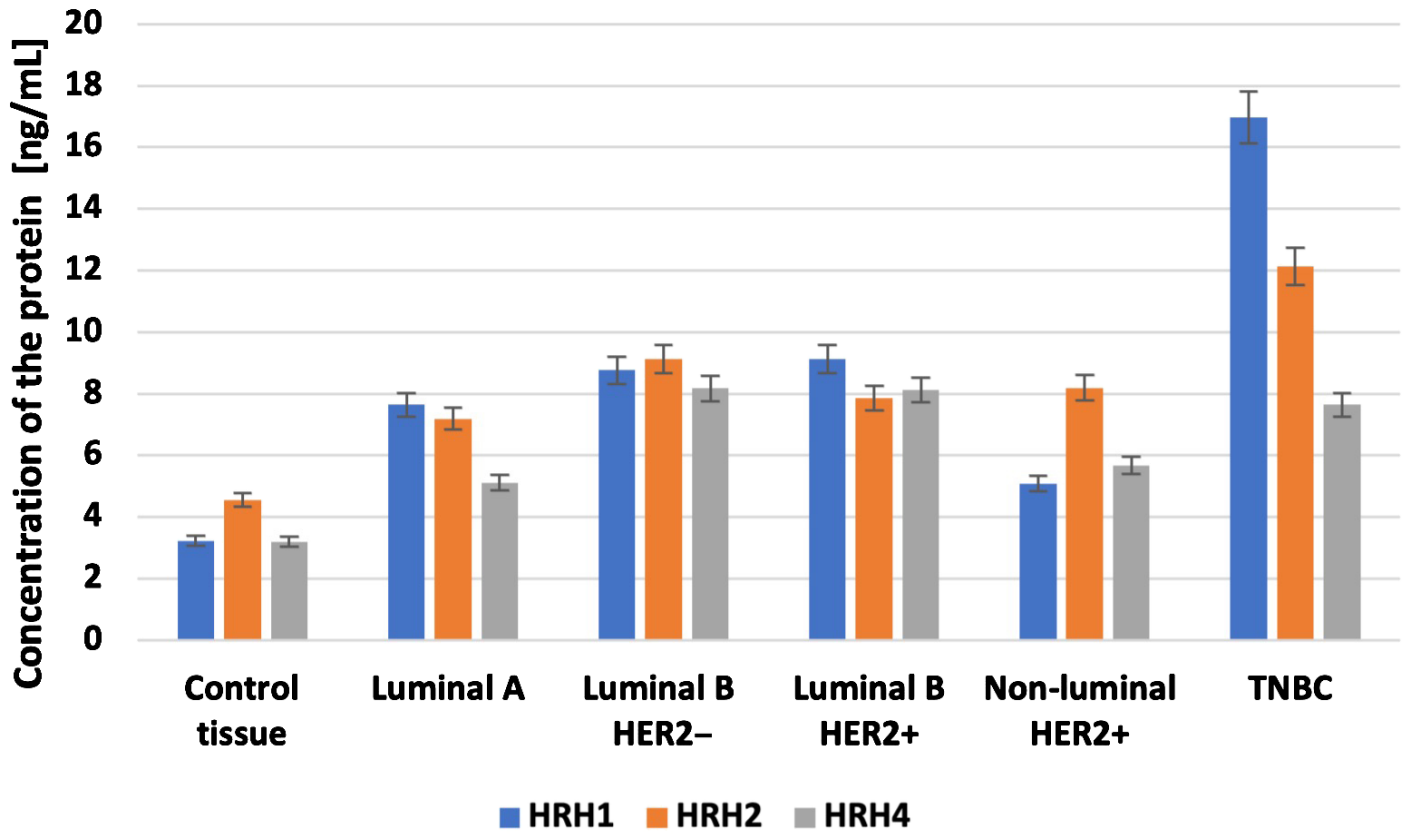
Relationship network for the proteins associated with the histaminergic system differentiation genes generated in the STRING database.

This study explored the intricate interactions between gene products related to the histaminergic system using the STRING Database (String Database 11.0). Proteins were represented as nodes, with edges indicating predicted functional associations based on experimental data, co-expression, gene neighborhood, text mining, and other evidence. Within this database, the proteins encoded by the analyzed genes formed a tightly interwoven network characterized by eight edges and 17 nodes (p < 0.001; average local clustering coefficient was 0.49, and average node degree was 0.941). The interactions are visually represented in a diagram (**Figure 8**), showing that HRH1, HRH2, and HRH4 are central to the network, indicating their significant roles in breast cancer pathology. These histamine receptors interact with proteins, such as HNMT and LYN, suggesting a complex regulatory mechanism involving histamine signaling in cancer progression. Endothelin 1 and its receptor (EDNRA) are connected to various proteins, including ADCYAP1 and HTR6, indicating their involvement in the signaling pathways that regulate vascular tone and cell proliferation. Additionally, ADA is linked to RGS4 and LYN, highlighting its role in purine metabolism and immune response modulation in the tumor microenvironment. The association of SLC23A2 with GABRB1 indicates potential interactions related to amino acid transport and neurotransmission in cancer cells. The prominent interactions of histamine receptors suggest their crucial role in breast cancer cell proliferation, survival, and angiogenesis, implying that therapeutic targeting of these receptors could disrupt these pathways and inhibit tumor growth. The involvement of endothelin signaling components underscores their contribution to cancer progression by promoting angiogenesis and cell proliferation, suggesting that targeting the endothelin pathway could be a viable cancer therapeutic strategy. The network connections of ADA suggest its role in modulating the tumor microenvironment, potentially affecting immune response and cell survival; modulating ADA activity could influence tumor progression and response to therapy. The association with GABRB1 indicates the significance of amino acid transport and neurotransmission in cancer cell metabolism and survival, highlighting potential new therapeutic avenues. Additionally, the genes were classified into 96 biological processes, five molecular functions, and five KEGG pathways.

### 3.6 Overall survival analysis of the selected histaminergic system differentiation genes

**Figure 9:**
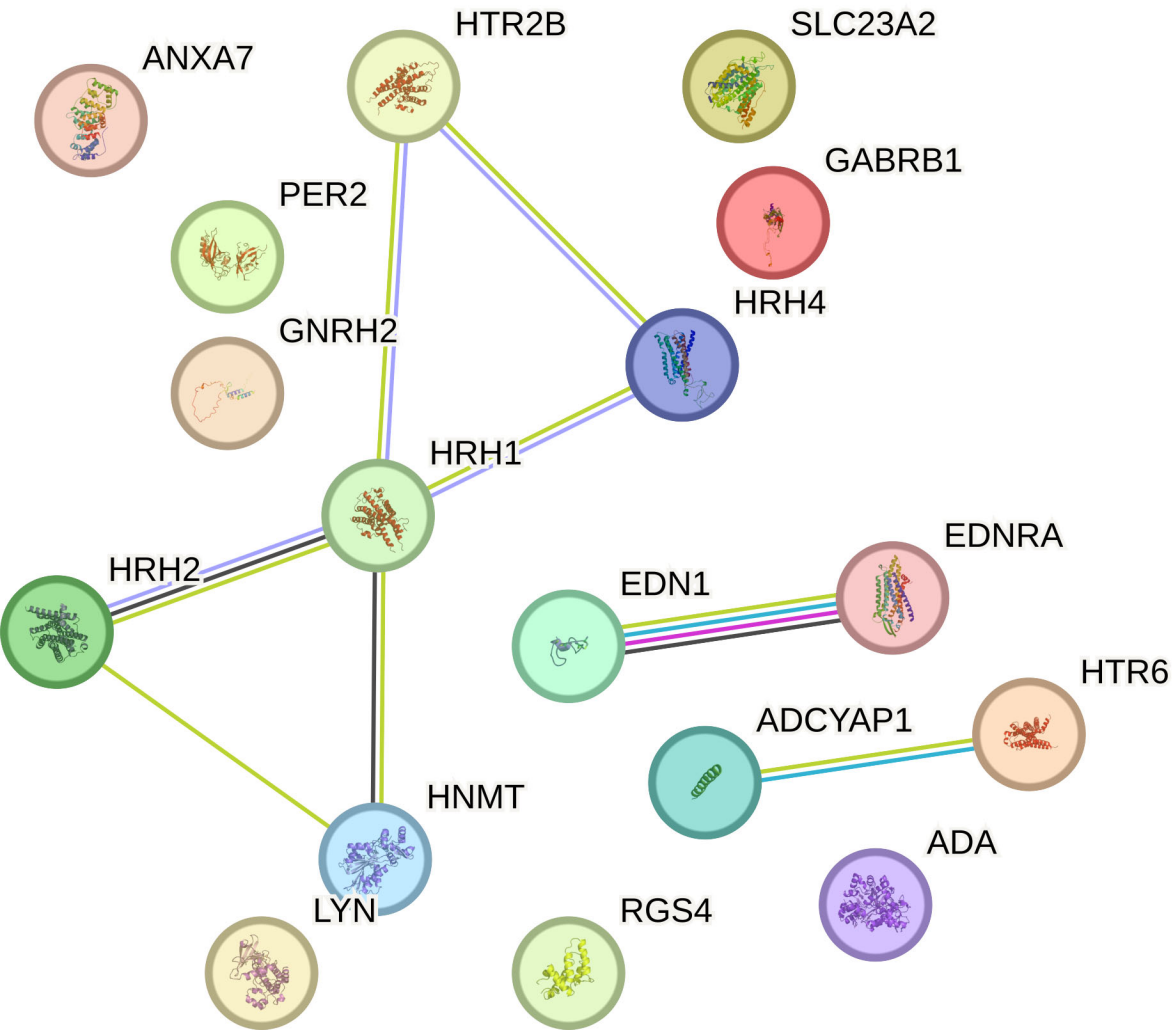
Overall survival analysis for luminal A subtype.

**Figure 10:**
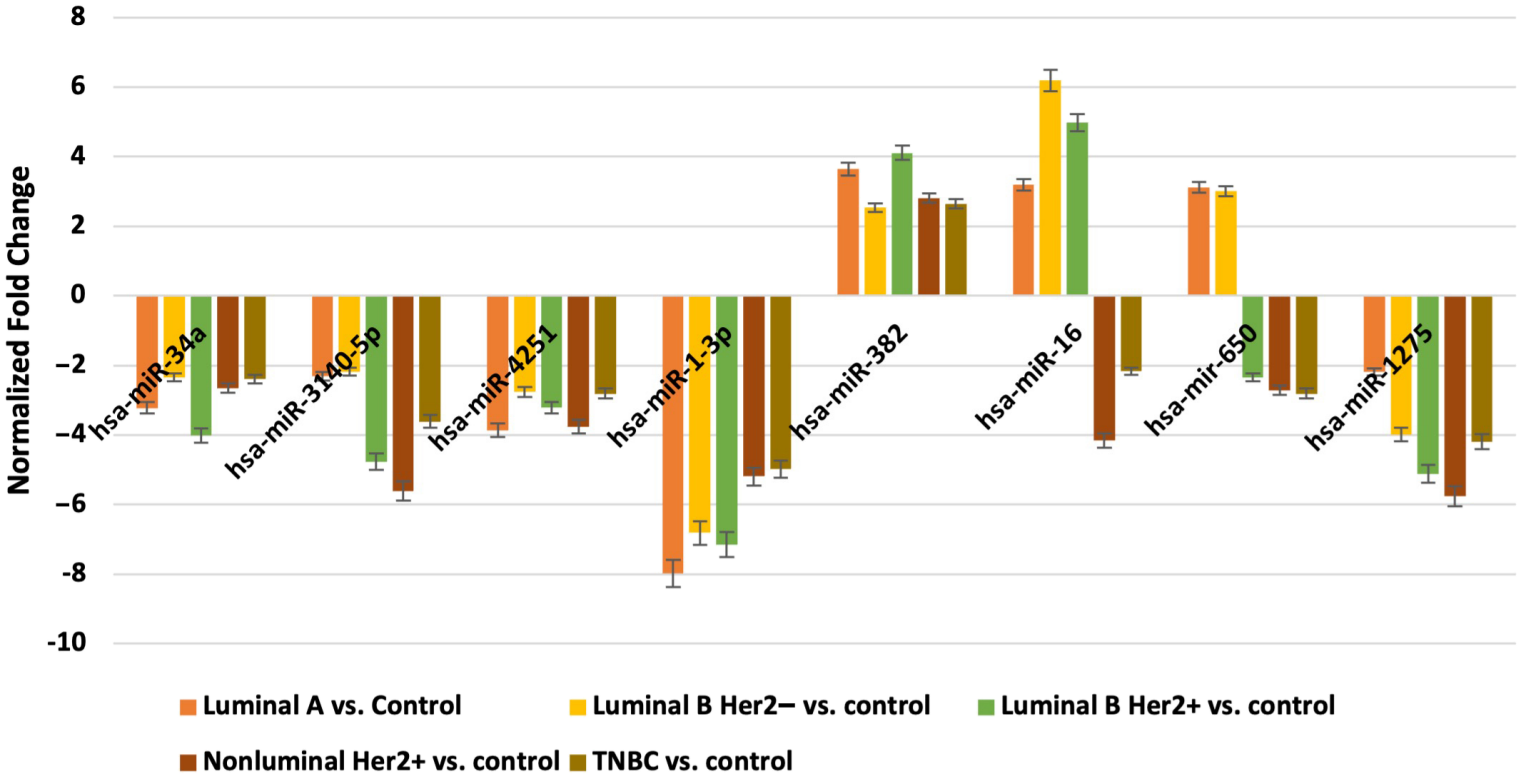
Overall survival analysis for luminal B HER2− subtype.

**Figure 11:**
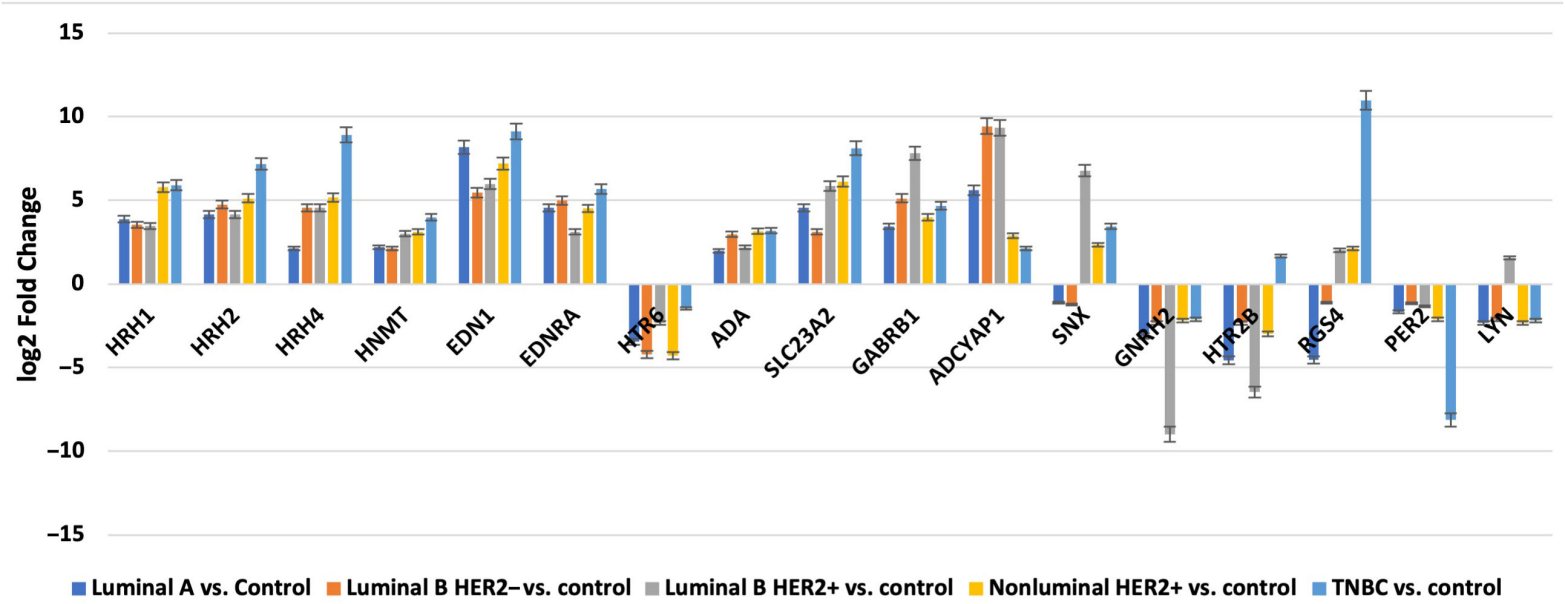
Overall survival analysis for luminal B HER2+ subtype.

**Figure 12:**
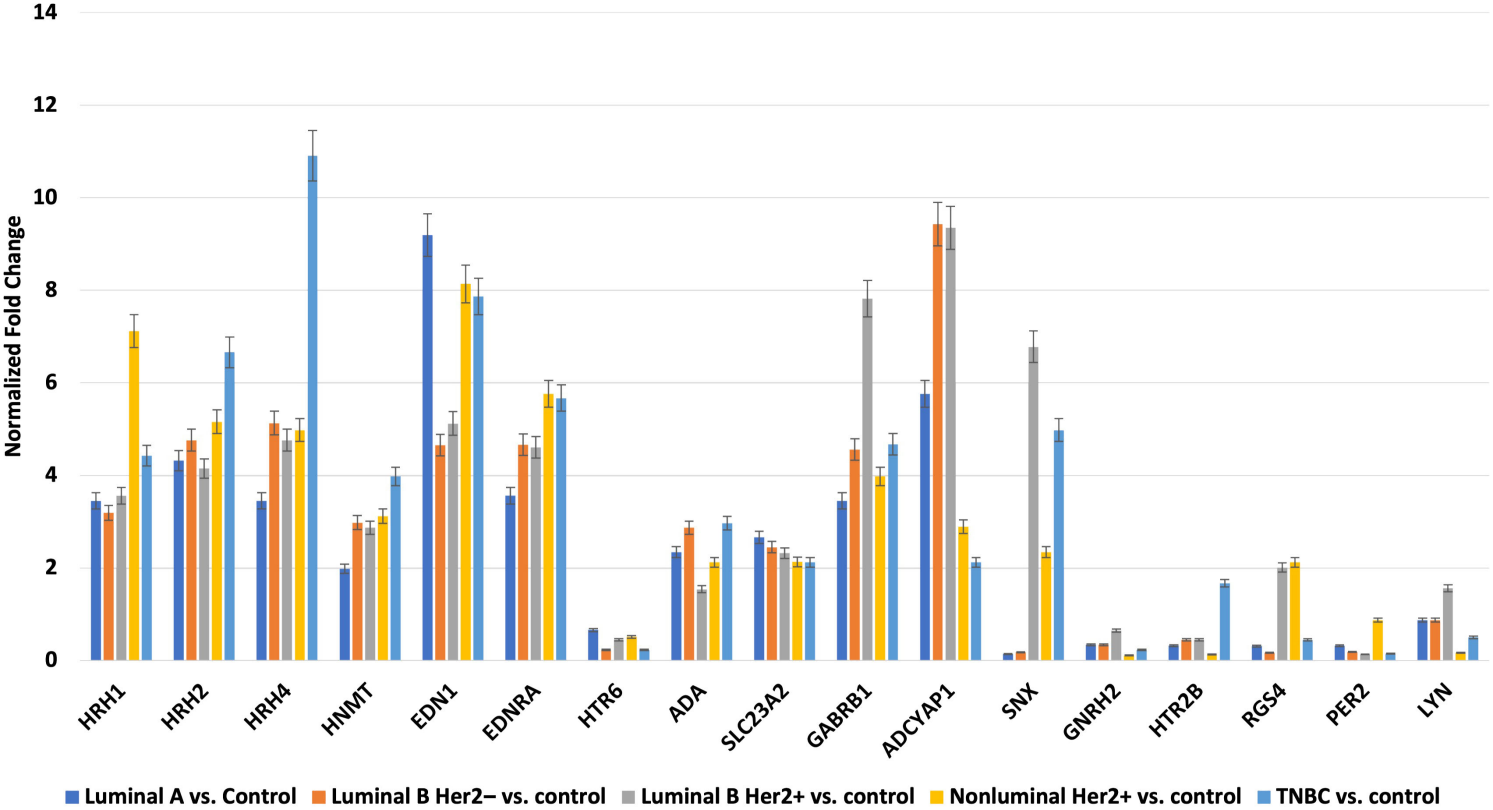
Overall survival analysis for non-luminal HER2+ cancers subtype.

**Figure 13:**
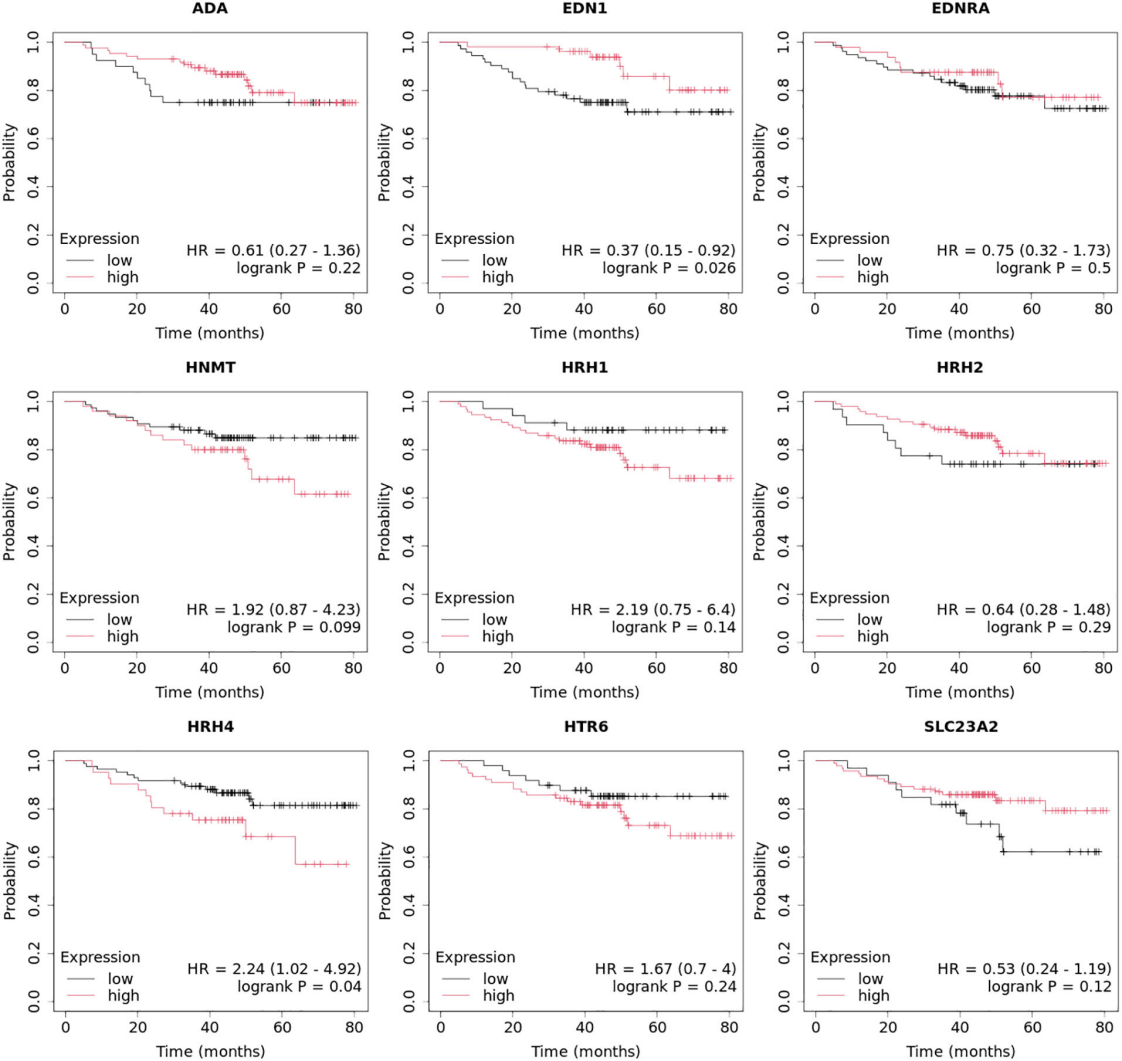
Overall survival analysis for TNBC subtype.

Overall survival analysis using the Kaplan–Meier plotter was performed for the studied mRNAs and presented for each breast cancer subtype (**Figures 9**–**13**).

In the case of the luminal A subtype, *EDNRA*, *HNMT*, and *SLC23A3* were important, and their low expression negatively affected overall survival (OS) (**Figure 9**).
In turn, high *END1* and *HRH1* expression, together with low *SLC23A3* activity, promoted worse OS in patients with luminal B HER2**−** (**Figure 10**).
In luminal B HER2+ samples, the expression of the analyzed genes was not important for OS (**Figure 11**).
High expression of *EDN1*, *HRH2*, and *HRH4* promoted worse OS in non-luminal HER2+ cancers (**Figure 12**).
In turn, low *EDN1* expression and high *HRH4* activity were important for OS in patients with TNBC (**Figure 13**).

## 4 Discussion

Considering the intricate nature of cancer, it is essential to develop effective therapies to address the various molecular components present within a tumor and their specific interactions within the tumor microenvironment (41, 42). Despite advances in cancer research in recent decades resulting in the development of new and enhanced anti-neoplastic drugs, anticancer therapy still yields adverse outcomes, such as inadequate response and significant toxicity (43).

The significance of assessing biogenic amine levels has been highlighted in various contexts. These include diagnosing Parkinson’s disease (44) and central nervous system infections, such as bacterial and viral meningitis [24]. Additionally, it aids in the early detection of neuroendocrine tumors (45), advancement of novel methods for examining neoplastic cells (46), and the development of therapies targeting biogenic amines, including antihistamines (47).

Histamine, a compound with pleiotropic effects, has potential as a therapeutic agent for cancer treatment. The administration of histamine dihydrochloride has received approval in Europe for the treatment of acute myeloid leukemia when combined with the immunotherapeutic agent IL-2, highlighting the potential of histamine in cancer therapy (48). Notably, endogenous histamine promotes murine LM2 breast tumor growth by suppressing anti-tumor immunity (49). Conversely, histamine treatment promoted dendritic cell maturation from monocytes, thereby reducing murine lymphoma growth developed with EL-4 cells (50). The presence of H1 and H2 receptors in normal and malignant human breast cancer tissues and cell lines has been extensively documented (51).

Therefore, the analysis of the histaminergic system is crucial because it is involved in regulating cell proliferation, which is a critical factor in the initiation and progression of tumors. Histamine, a primary mediator of this biological process in various cancer types, influences cell growth and division. Understanding the role of the histaminergic system in cancer development may lead to the identification of new therapeutic targets and development of more effective cancer treatments (31–33).

Our molecular analysis showed that of the nine genes that differentiated breast cancer biopsy specimens, regardless of the subtype from controls, only *ADA* and *SLC23A2* expression was silenced in the tumor samples. The expression of the remaining seven mRNAs was significantly higher in tumor tissues than in control tissues (p < 0.05).

Histamine, which is synthesized by L-histidine decarboxylase, exerts its effects through paracrine or autocrine mechanisms, contributing to various processes that foster tumor growth. These include modulation of the immune response and regulation of the proliferation, angiogenesis, differentiation, apoptosis, and migration of cancer cells. Its multifaceted impact is facilitated by its interaction with H1–H4 receptors, whose expression varies across different tissue types (52). Activation of the H1 receptor triggers the activation of phospholipase C and a subsequent increase in intracellular Ca^2+^ levels, which are potentially regulated by calmodulin (53, 54).

Our findings are consistent with those of Fernández-Nogueira et al. (52), who identified the upregulation of H1 receptors in basal and HER2-enriched breast cancers, correlating with poorer prognosis. They proposed a therapeutic strategy involving terfenadine, a H1 receptor antagonist that inhibited the migration of basal breast cancer cells and induced apoptosis (52). Similarly, Zhao et al. observed increased H1 receptor expression in hepatocellular carcinoma, which led to enhanced cell cycle progression and suppressed apoptosis, thereby promoting metastasis. Terfenadine treatment in a hepatocellular carcinoma xenograft model inhibited tumor growth (55). Matsumoto et al. reported H1 receptor overexpression in cisplatin-resistant HeLa cells, where antagonists, such as cloperastine, selectively killed tumor cells (56). Although desloratadine and loratadine hold promise for cancer therapy, further investigations are warranted (25). Another H1 receptor antagonist, astemizole, when combined with histamine, induces autophagy and apoptosis by targeting p53-dependent crosstalk in human MCF-7 breast cancer cells (57). These findings support the therapeutic potential of targeting H1 receptors in breast cancer.

In turn, activation of the histamine H2 receptor leads to an elevation in intracellular cyclic adenosine monophosphate levels, with effects often exerting actions opposing those mediated by the H1 receptor across various biological processes, such as the immune response (49, 58). Interestingly, Gao et al. observed improved overall survival in colon cancer patients with H2 receptor overexpression (59). Despite promising preclinical data, clinical trials investigating H2 receptor antagonists for breast cancer have yielded inconclusive results (27, 30, 60). Recent studies have shown that H2 receptor blockers, such as cimetidine and famotidine, are not associated with an increased risk of breast cancer, whereas ranitidine users have a higher risk of developing ductal carcinoma (61). Based on available evidence, treatment with H2 antagonists does not seem to be beneficial from a therapeutic perspective.

Similarly, the H4 receptor has emerged as a potential therapeutic target because of its role in anti-tumor immunity, as demonstrated in TNBC (28). Reduced levels of this receptor have been noted in colorectal cancer (62) and oral tongue squamous cell carcinoma (63), whereas overexpression of the H4 receptor inhibits the proliferation of cholangiocarcinoma (64) and esophageal squamous cell carcinoma (65). Using a TNBC model, Sterle et al. found that H4 receptor deficiency reduced tumor growth and metastasis, highlighting its potential as a therapeutic target (28). Thus, variations in histamine metabolism, distinct tumor microenvironments, and availability of histamine receptors may influence outcomes induced by histamine receptor ligands (31, 66, 67).

Another gene found to be overexpressed in tumor tissues is *HNMT*; however, a study on a Chinese Han population underscored the significance of histidine decarboxylase gene (*HDC*) polymorphisms, rather than those of the *HNMT* gene in breast cancer, further highlighting HDC’s importance in this disease (68).

Our study revealed significant connections between the histaminergic system and genes involved in angiogenesis, such as EDN1 and EDNRA. The role of endothelin signaling in cancer progression warrants further exploration, particularly regarding the potential use of EDNR antagonists in cancer therapy (52, 53).

Additionally, the overexpression of ADA and SLC23A2 in tumor tissues suggests that these genes could be interesting therapeutic targets (69).

Our microarray analysis also showed overexpression of *ADA* mRNA in tumor tissues compared to normal tissues, which is consistent with the observations of other researchers (70, 71). Moreover, a correlation between ADA expression, breast cancer stage, and metastatic potential has been reported (72), making restoration of the normal ADA expression pattern an interesting therapeutic target.

One of the last mRNAs that differentiated cancer samples independent of subtype from control samples, for which a decrease in transcriptional activity was demonstrated in cancer tissues, was *HTR6.*

The HTR6 receptor, a 5-hydroxytryptamine 6 receptor, interacts with the Gsα protein, leading to the activation of cAMP production (73). This receptor is predominantly present in the central nervous system and is involved in regulating various functions, such as cognition, appetite, mood, and epileptic activity (74). Its dysregulation has been linked to conditions, such as schizophrenia and Alzheimer’s disease (75). Additionally, according to our current findings, a study by Jinhua et al. revealed decreased HTR6 expression in colon cancer, suggesting a potential role in tumor suppression and recurrence (76), particularly in TNBC samples. Our observations regarding reduced *HTR6* expression in tumor tissues are consistent with the observations made by Zhang et al. (77), who after performing immunohistochemical staining, suggested that HTR6 may have an inhibitory effect on breast cancer progression (77). Moreover, Zhang et al. found that sertindole, an antipsychotic drug (sertindole), has a pro-apoptotic effect on breast cancer cells (78). Moreover, in breast cancer patients, compared to normal breast tissue and para-tumor tissues, HTR6 expression was increased in *in situ* breast cancer, but decreased in invasive breast cancer, and almost no expression was found in distal and lymphatic metastases, showing a trend wave during the development of breast cancer. Therefore, it appears that HTR6 has a dual action, depending on the biological context (77, 78). These observations are also consistent with the study by Zhan et al., which showed no statistically significant differences in the expression of HTR1, HTR3, HTR5, and HTR6 between breast adenocarcinoma and normal samples. These authors also noted that HTR6 expression in high-grade breast cancer was lower than that in less invasive cancers, which requires further studies considering our results (79).

The role of miRNAs in regulating the histaminergic system is a fascinating area of study (80). Our predictive analysis revealed several potential interactions between the miRNAs and components of the histaminergic system. First, certain miRNAs, such as hsa-miR-34a, hsa-miR-3140-5p, and hsa-miR-4251 may be involved in modulating the expression of histamine receptors, such as *HRH1* and *HRH2*. Conversely, other miRNAs, such as hsa-miR-1-3p, may play a role in regulating the expression of *HRH4* and *EDN1*.

Moreover, expression of key enzymes and receptors within the histaminergic system, such as *HNMT* and *EDNRA*, may be influenced by specific miRNAs. For example, hsa-miR-382 may potentially regulate *HNMT* expression, whereas both hsa-miR-34a and hsa-miR-16 may be involved in the modulation of *EDNRA* expression. Additionally, miRNAs, such as hsa-miR-650 and hsa-miR-1275, interact with components of the histaminergic system, possibly affecting the expression of genes, such as *HTR6*, *ADA*, *SLC23A2*, and *HRH1*.

The present study has several limitations. First, the sample size, especially for subtypes, such as HER2+ and TNBC, may constrain the generalizability of our findings, indicating the need for a larger cohort to enhance statistical robustness. Second, the focus on the Polish female population limits the diversity of the patient cohort, potentially affecting the wider applicability of the results. Third, although microarray analysis and qRT-PCR are pivotal for assessing gene expression patterns, their inherent limitations in capturing the detailed intricacies of gene regulation suggest the need for alternative omics methodologies, such as RNA sequencing, for a more comprehensive understanding. Additionally, the concentration of proteins was evaluated solely using ELISA. Incorporating immunohistochemistry and western blotting would be crucial for a more thorough protein expression analysis.

Despite these limitations, this study has several strengths. This multifaceted approach, encompassing gene expression profiling, miRNA analysis, and protein quantification via ELISA, allowed for an in-depth examination of the histaminergic pathways in breast cancer. The inclusion of various breast cancer subtypes increases the relevance and applicability of our findings across diverse patient populations. Additionally, rigorous adherence to standardized protocols for gene expression and miRNA analyses ensured the reliability and reproducibility of our results. Utilizing reputable databases for miRNA target prediction helped elucidate the potential regulatory mechanisms underlying histamine-mediated gene expression alterations.

Overall, these findings suggest a complex network of interactions between miRNAs and the histaminergic system, highlighting the intricate regulatory mechanisms governing histamine signaling in the body. Further studies in this area could provide valuable insights into the role of miRNAs in health and disease, especially in conditions involving histamine dysregulation. Moreover, the analysis showed that the selected miRNAs did not exhibit complete complementarity with their target mRNAs. The crosstalk among miRNAs, the histaminergic system, and breast cancer represents a complex regulatory network with significant implications for cancer biology and therapeutic development.

Our analysis confirms the intricate nature of the histaminergic system and its crucial role in the development and progression of breast cancer. Molecular analyses indicated that the selected mRNA and miRNAs could serve as promising molecular markers and potential therapeutic targets.

## Data Availability

The data used to support the findings of this study are included in the article. The data cannot be shared due to third-party rights and commercial confidentiality.
