# Supplementary material for: Expression profile of messenger and micro RNAs related to the histaminergic system in patients with five subtypes of breast cancer
Source: Front Oncol. 2024 Aug 29;14:1407538. doi: 10.3389/fonc.2024.1407538 (PMC11390352; doi:10.3389/fonc.2024.1407538)
Supplement: Supplementary file 1 [file Table1.docx]

Supplementary Material

**Supplementary Table 1.** Sequence of primers used in RTqPCR

| **mRNA** | **Nucleotide sequence** | |
| --- | --- | --- |
| *HRH1* | Left | 5′-TATGTTTAAGTGGTTATTGGGTTGT-3′ |
|  | Right | 5′-ACCAAAACTCAAATCTTAATACAAT-3′ |
| *HRH2* | Left | 5′-ATAGTTTTGGTTTTAGTTTTGTTGT-3′ |
|  | Right | 5′-CAAAACATATTCATATCCCTTCACT-3′ |
| *HRH3* | Left | 5′-TTTTATATTGGGTTTAGTAGGGTGATAT-3′ |
|  | Right | 5′-CTCCCAACTCAAAATAACTAATCCA-3′ |
| *HRH4* | Left | 5′-TTAGTATTTTGGGAGGTTAAGGTG-3′ |
|  | Right | 5′-TTCTATTACCAAACTAACAAACTCC-3′ |
| *HNMT* | Left | 5′-GTTTGTAGTTAAGATATTGAATTTTGA-3′ |
|  | Right | 5′-AAAATCATCCTAAAAAAAACATA-3′ |
| *EDN1* | Left | 5′-AGGTTTGAAATTTTGTATTTTTTTT-3′ |
|  | Right | 5′-CCACCTTACTAAAACTACCCCTACA-3′ |
| *EDNRA* | Left | 5′-ATAAATGTATGAGGAATGGTTTTAA-3′ |
|  | Right | 5′-AAAAAAAACAACTTACAAAAAAATAC-3′ |
| *SLC23A2* | Left | 5′-GTGTGGGTAAAGGGAATAAATTATT-3′ |
|  | Right | 5′-ACCACAAAACACAACAAAAACTATC-3′ |
| *HTR6* | Left | 5′-TTCCTGGTGTCGCTCTTCAC-3′ |
|  | Right | 5′-CACAAGGACAAAAGGCAGGC-3′ |
| *GABRB1* | Left | 5′-AGTAGAATTGTATTTTGGAGATTGA-3′ |
|  | Right | 5′-AAACAAAAACTTAATAACAATCATA-3′ |
| *ADCYAP1* | Left | 5′-AAGTATTTGTAGTTGTTTGTGGTTTG-3′ |
|  | Right | 5′-TATAACTATCCATAAAAATCCCATC-3′ |
| *SNX* | Left | 5′-GGTATAATGATTGTAGGTGATATGA-3′ |
|  | Right | 5′-TCCTTAACAAAAAACTCTTTCAAT-3′ |
| *GNRH2* | Left | 5′-GTATGTTTTGGGAGGGTAGGATTAT-3′ |
|  | Right | 5′-TTAAAAAATAACAAAACAAAACAAA-3′ |
| *HTR2B* | Left | 5′-GATGAAATATTTTGTTTGTTATTGG-3′ |
|  | Right | 5′-AAACCTTTAAAACTCTCTATTCATT-3′ |
| *RGS4* | Left | 5′-TGTATTAGGGAAGAGATAAGTTGG-3′ |
|  | Right | 5′-TTAACCAAATCAAAATAAAATCAAA-3′ |
| *PER2* | Left | 5′-GATAGTTTGGGGGTTAGTTTTTTT-3′ |
|  | Right | 5′-CAATACCAACAAAATCAAATACATA-3′ |
| *LYN* | Left | 5′-AACTCAAGTCACCGTGGAGC-3′ |
|  | Right | 5′-TGGAACTGGCCTTTGCTGTT-3′ |

ADCYAP1A, adenylate cyclase activating polypeptide 1; SNX, Sorting Nexin 1; *HNMT*, histamine N-methyltransferase; *HTR6*, 5-hydroxytryptamine receptor 6; *GNRH2*, gonadotropin-releasing hormone 2; *HTR2B*, hydroxytryptamine receptor 2B; *HRH1*, histamine receptor 1; *HRH2*, histamine receptor 2; *HRH4*, histamine receptor 4; *HTR6*, 5-hydroxytryptamine receptor 6; *GABRB1*, gamma-aminobutyric acid (GABA) A receptor, alpha 1; *EDN1*, endothelin 1; *EDNRA*, endothelin receptor type A; *SLC223A2*, solute carrier family 22 member 3; *LYN*, LYN proto-oncogene; *ADA*, adenosine deaminase; *RGS4*, regulator of G-protein signaling; *PER2*, period circadian clock2; LYN, LYN proto-oncogene, Src family tyrosine kinase
